# Cusp deflection and fracture strength of root canal filled premolars with two access cavities designs (Conservative vs Traditional)

**DOI:** 10.4317/jced.59460

**Published:** 2022-09-01

**Authors:** Al-alaa J. Mowlood, Ahmed H. Ali, Anas F. Mahdee

**Affiliations:** 1 BDS, Oral and dental science, College of Dentistry, University of Tikrit, Salah Addin, Iraq; 2 BDS, MSc and PhD (UK), Aesthetic and restorative dentistry department, College of Dentistry, University of Baghdad, Baghdad, Iraq

## Abstract

**Background:**

This study evaluated two endodontic access designs (Conservative (Cons) vs Traditional (Trad)) of class I and class II cavities on cusp deflection (CD) and fracture strength (FS) of root canal filled maxillary premolars.

**Material and Methods:**

Seventy-two sound maxillary first premolars were included in this study; Teeth were randomly assigned into nine groups (n=8), a positive control group where teeth left sound and the other eight according to the access cavity designs (Cons class I, Trad class I, Cons class II and Trad class II). After access preparation, teeth were endodontically-treated. Four groups were restored with FiltekTM Bulk-Fill composite. While the other four groups were left without coronal restoration as negative controls for fracture strength. Following thermocycling (500 cycles), CD values were recorded for the restored teeth at the following intervals, after cavity preparation, 15 min after restoration and after thermocycling. The samples were then subjected to fracture using a universal testing machine. The data were analyzed using one-way ANOVA and Tukey Post-Hoc. Statistical significance was set at *p*<0.05.

**Results:**

There was a significant difference between groups at 15 min after restoration (*p*<0.000) and there were no differences after cavity preparation and after thermocycling (*p*>0.05). At 15 min after restoration, the CD value was significantly higher in Trad class II than in other types of cavities (*p*<0.05) and there was no difference between Cons and Trad class I (*p*>0.05). In each group, the CD value was significantly higher 15 min after restoration compared to that after cavity preparation and after thermocycling in all groups. The highest FS was recorded for the control group (1240 N), while the lowest was for the Trad class II not restored group (472.8 N). One-way ANOVA test showed a highly significant difference between groups (*p*<0.000) and there were no significant differences in FS between the Cons vs Trad access cavity designs in class I and class II cavities, respectively.

**Conclusions:**

Cusp deflection increased by the increase in the size of cavity preparation and stress relaxation tends to occur after thermocycling. Moreover, the conservation of the endodontic access cavity could improve the resistance of the tooth to fracture compared to its traditional counterpart but not to a statistically significant point.

** Key words:**Conservative, traditional, endodontic access cavity, cusp deflection, fracture strength.

## Introduction

Access cavity, with its different designs, is the first step to be achieved during endodontic treatment. This cavity enables the identification of root canals, insertion of instruments, delivery of disinfecting irrigants and placement of the definitive root filling (1). However, Endodontically-treated teeth are more vulnerable than vital teeth to fracture during function (2). This failure is induced by a variety of causes, the most significant one is the loss of dental structure, which in part is affected by coronal access cavity design (3).

Traditional access cavity designs for various tooth types have remained relatively unaltered for decades, with only slight changes addressing convenience form and extension for prevention (4). Consequent tooth structure reduction, coronal to the pulp chamber, along chamber walls, and around canal orifices, may compromise the tooth’s resistance to fracture under functional loads (5). Also, a study has found that the remaining tooth structure has a significant association with the outcome of endodontic retreatment (6). As a result, conservative access cavity design was suggested to preserve as much tooth structure as possible (7), inspired by the concept of minimally invasive dentistry.

Cusp deflection is a common biomechanical phenomenon that happens in teeth restored with composite resin materials and is caused by the interaction between the materials’ polymerization stress and the remaining tooth structure compliance after preparation (8). Cusp deflection can lead to occlusion changes, enamel cracking, and tooth fracture (9,10).

Indeed, these newly emerging conservative access modalities are often more technically demanding than their traditional ones. They also make procedures of canal detection, cleaning, and shaping more difficult, as well as increase the risk of iatrogenic complications (11-13). In this context, several studies have been performed to investigate if such conservative access strategies have an impact on root canal treatment outcomes and root filled teeth fracture resistance (1,4,11,13-15), and showed contradicting findings. Moreover, no study evaluated the relation of access design with cusp deflection with different classes of cavities. Therefore, this study aimed to evaluate the effect of Conservative vs Traditional access cavity designs in class I and class II cavities on the cusp deflection and fracture strength of root-filled premolars. The null hypotheses stated that there are no differences in cusp deflection and fracture strength of premolars with different access cavity designs.

## Material and Methods

-Samples Selection

The sample size was calculated using G*Power 3.1.9.4 (Heinrich Heine University, Düsseldorf, Germany) based on the results from a previous study (14) with an effect size of 0.83, power 0.9, α error 0.05, thus requiring six teeth for each group. Eight teeth were assigned for each group in this study. A total of seventy-two teeth were selected from a group of freshly extracted, intact maxillary first premolars extracted for orthodontic reasons and were collected after ethical approval (project no. 289521, ref. number 289). Teeth were examined using a magnifier (10x) for any signs of caries, visible crack, restoration, or attrition to be excluded. To reduce the confounding variables, the selected teeth had comparable sizes which had been assessed by measuring the buccolingual, mesiodistal, and occlusocervical dimensions in millimeters using a digital vernier. The accepted difference within each of these dimensions was no more than 5% of the determined means. Teeth then were disinfected in 0.1 % thymol solution for 48 hrs, before storing them in distilled water at room temperature.

Then the root part of each tooth was embedded within an acrylic block at the levels of cemento-enamel junction by using a pre-fabricated silicon mold. This was performed to facilitate the handling of samples during experimental procedures.

-Experimental groups

The teeth were randomly divided into four main groups (n=16) and one positive control group (n=8) as follows:

Positive control: sound teeth without cavity preparation.

**Figure 1:**
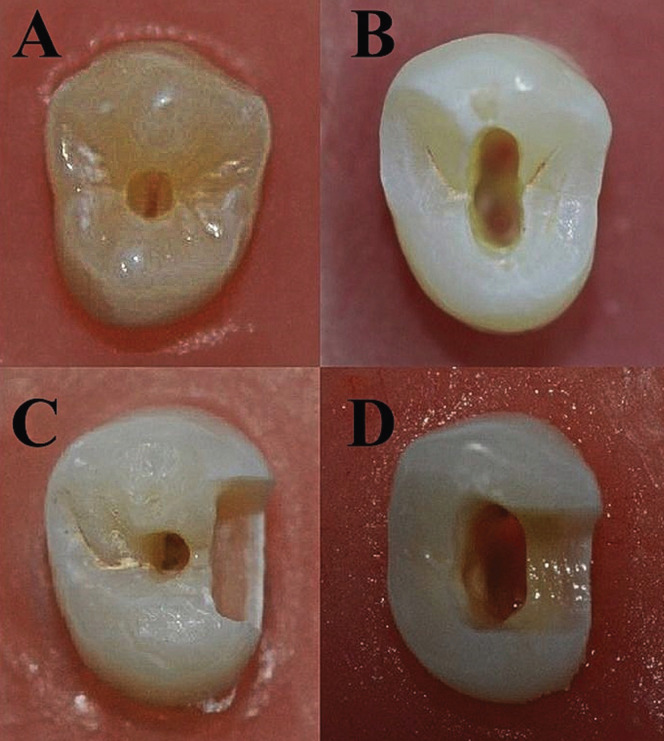
Access cavity designs performed in this study: A: Cons class I B: Trad class I, C: Cons class II, D: Trad class II.

Cons class I: The access cavity was performed to the pulp chamber occlusally through the central fossa with dimensions equal to the diameter of the round diamond bur (1.2 mm in diameter) (Komet, Lemgo, Germany, LOT: 00213371), without expanding the cavity in buccolingual or mesiodistal directions to avoid complete removal of the pulp chamber roof (16), as shown in Fig. 1.

Trad class I: Access cavity to the pulp chamber performed occlusally through the central fossa using round diamond bur (Komet, Lemgo, Germany, LOT: 00213371), before complete de-roofing of the pulp chamber using endo Z bur (Dentsply Sirona Endodontics, Ballaigues, Switzerland) to reflect the established literature’s traditional standards (16), as shown in Fig. 1.

Cons class II: Mesial box (4mm width x 4mm depth x 2mm length) was prepared using flat ended diamond fissure bur (Komet, Lemgo, Germany, LOT: 883979), and access cavity to the pulp chamber was performed occlusally without connection with the box and without complete deroofing of the pulp chamber similar to Cons class I group, as shown in Fig. 1.

Trad class II: Mesial box (4mm width x 4mm depth x 2mm length) was prepared using flat ended diamond fissure bur (Komet, Lemgo, Germany, LOT: 883979) with continuous communication to the access cavity and complete deroofing of the pulp chamber, as shown in Fig. 1.

The endodontic access cavity was performed by using a high-speed handpiece (NSK, Tochigi, Japan) mounted on a modified dental surveyor under profound water cooling. The used burs were replaced every four preparations to ensure high cutting efficiency. The cavosurface angle was kept at 90o. A graded periodontal probe was used to determine the dimensions of the prepared cavity which were verified using a dental vernier at different points. After completing access cavities, root canals within each tooth were negotiated with stainless steel K- files size 10 and 15, working length determined before root canals instrumented up to size 25 taper 0.04 (F360®, Komet, Legmo, Germany), then irrigated and obturated with AH plus (Dentsply Sirona Endodontics, Ballaigues, Switzerland) and single gutta percha cone obturation technique (Komet, Legmo, Germany) in a standardized procedure. Then the samples within each of the four main groups were further divided into 2 subgroups (n=8); depending on being restored or left without restoration.

-Restorative procedure

Samples to be restored within each group were etched with a selective-etch technique using 37% phosphoric acid (Super etch, SDI, Victoria, Australia, LOT: 201270) and placed on enamel only for 30 seconds (17). Then cavities were washed thoroughly with distilled water and dried with an air syringe before applying two separate coats of Single Bond Universal (3M ESPE Oral Care, St. Paul, USA) and light cured for 20 seconds, according to manufacturer’s recommendations, with a LED light curing device (Curing Pen, Eighteeth, China, SN EL1C0926A011). A LED radiometer (Coxo, Guangdong, China) was used to monitor light intensity, which should be 1000mw/cm2, before curing each group.

 Cavities up to 5mm were restored by one increment of FiltekTM Bulk-Fill composite (3M ESPE Oral Care) according to the manufacturer’s instructions and cured for 20 sec. For class II cavity groups, the SuperMat® Matrix system (Kerr , Bioggio, Switzerland) was used to fit the band and tighten it around each sample, the excessive tightness was avoided to prevent the effect of the band on results.

After 24 hours, all samples were subjected to thermocycling in the cold (5oC) and hot (55oC) water bath for 500 cycles, 30 seconds per cycle (ISO TR 11405). All endodontic and restorative procedures were performed by a single operator.

-Intercuspal distance measurement and cusp deflection

Measurements were performed on images captured using a computerized digital microscope (Q-Scope® QS.90200-P, Netherlands) at a magnification of 150x in combination with Image J software (ImageJ bundled with Java 1.8.0_172, USA). Two reference points (orthodontic wire fragments) were bonded as close as possible to the cusp tips of samples within restored groups to measure the intercuspal distance (ICD) accurately. The ICD before cavity preparation was measured and considered as the baseline measurement. Then cusp deflection (CD) after cavity preparation, 15 min after restoration, and after thermocycling (CD 1, 2 and 3, respectively) was calculated by subtracting the ICD measurements during restorative phases (after cavity preparation, 15 min after restoration, and after thermocycling, respectively) from the baseline ICD measurement. CD measurements were performed for the restored teeth groups only.

-Fracture strength

All specimens were subjected to compressive axial loading in a a computer-controlled universal testing machine (Gester, GT-K03B, China) until fractured. The 1 mm/minute crosshead speed of a steel bar with a ball at its end (5 mm in diameter) was used to apply the load. The maximum load a sample can withstand before fracture was recorded in Newton (N). Samples were then investigated under a magnifier (10x) to assess the fracture pattern. The fracture line above the cemento-enamel junction was considered a restorable fracture, while that extended below the CEJ was considered a non-restorable fracture as shown in Fig. 1. Fracture strength measurements were performed for all teeth (restored and not restored).

-Statistical Analysis

The data were represented using descriptive statistics including means and standard deviations. Normality and homoscedasticity of CD and FS data were checked using Shapiro-Wilk and Levene’s tests respectively. One-way ANOVA with Tukey Post-Hoc test was used in analyzing the results (comparing CD1, CD2 and CD3 between the groups and within each group). Also, one-way ANOVA and Tukey Post-Hoc tests were conducted to compare fracture strength between different groups. Statistical analysis was performed using SPSS software version 26 (SPSS Inc., Chicago, IL, USA).with significance set at *p*<0.05.

## Results

Means and standard deviations of CD values within the groups of the restored teeth are listed in Table 1. The highest values were for CD2 (15 min after restoration within each group) while the lowest values were for CD3 (after thermocycling). CD values data were normally distributed (Shapiro-Wilk test p˃0.05) and the data were homogenous (Levene’s test p˃0.05). ANOVA test revealed no statistically significant difference between groups (*p*>0.05) except for CD2 values (*p*<0.05). Tukey test shows statistically lower CD values were recorded for the Cons and Trad class I groups compared to the Cons and Trad class II groups. Also, a statistically lower CD value in Cons class II compared to Trad class II was recorded, as shown in Table 1.

One-way ANOVA was used to compare CD values within the same restored group. The results showed a statistically significant difference in CD values within each group (*p*<0.05). Tukey Post-Hoc test revealed statistically significant differences between CD1 vs CD2 and CD2 vs CD3 values, while no significant differences were detected between CD1 vs CD3 values within each tested group, as shown in Table 1.

**Table 1:**
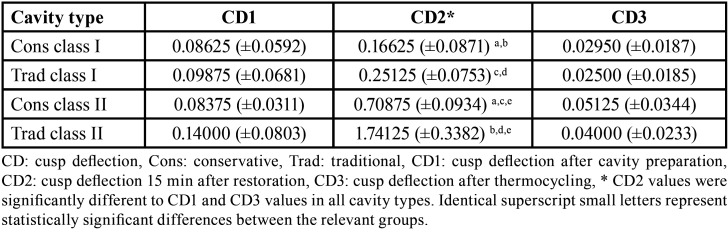
Mean and standard deviation (µm) of cusp deflection (CD) of different cavity types at different time intervals.

The mean and standard deviation of fracture strength values of restored and not restored teeth in all groups were listed in Table 2. The highest fracture strength value was 1240 N for the sound teeth group and the lowest was 472.8 N for the Trad class II not restored group. The fracture strength data were normally distributed (Shapiro-Wilk test *p*>0.05) and the data were homogenous (Levene’s test *p*>0.05). One-way ANOVA test of fracture strength data showed a statistically highly significant difference between groups (*p*<0.000). Tukey Post-Hoc test revealed that there was no significant difference between Cons and Trad designs in class I or class II cavities, respectively. However, there was significantly higher fracture strength in class I restorations compared to Trad class II restorations, as shown in Table 2.

**Table 2:**
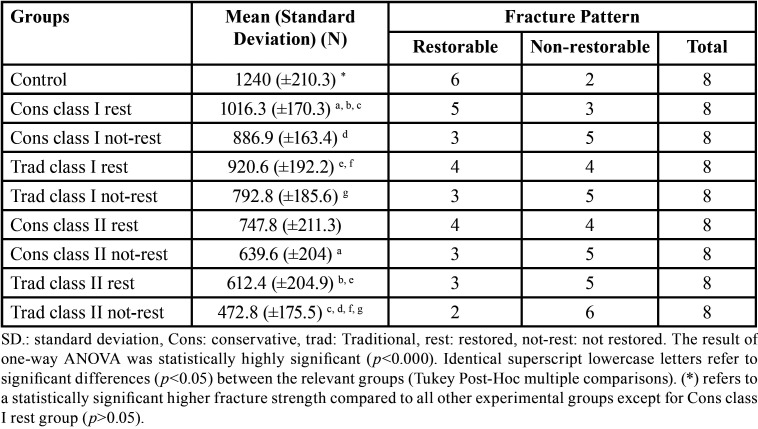
Mean and standard deviation of fracture strength of teeth in the study groups and their patterns of fracture.

Regarding the fracture pattern of the samples, the recorded results are shown in Table 2. The highest frequency of Non-restorable fractures was recorded in Trad class II, while the lowest was in the control group.

## Discussion

Conservative access cavity preparation was suggested to preserve as much tooth structure as possible (18), where recent research has attributed tooth structural loss to the majority of teeth extracted due to fracture (5). Conservative access cavities were firstly proposed by Clark and Khademi in 2010 (7). However, there are no specific guidelines for preparing the conservative access cavity. The goal is to maintain as much tooth structure as possible while still locating the canal orifices (15). However, an attempt has been made in a recent review to classify minimal access cavity preparations according to a universal nomenclature to reduce differences in guidelines among both clinicians and researchers (16).

In this study, teeth in the conservative access cavity design groups were accessed at the central fossa and only extended apically, preserving a significant portion of the pulp chamber roof and lingual shelf (14). Clinically, however, these techniques are primarily used on intact teeth that will be treated endodontically. This clinical scenario does not appear to occur frequently, accounting for only 8% of the cases reported by Plotino *et al*. in 2016 (14). On the other hand, the teeth accessed by the traditional access cavities were prepared following the conventional guidelines (19), in which straight-line access to the canal orifice is to be achieved by reducing coronal interferences and removing all pulp chamber roof with smoothly divergent walls. The conservative class II proximal-occlusal cavity scenario implemented in this study can be applied to intact teeth with an initial proximal carious lesion in teeth that needs endodontic treatment, where the preservation of tooth structure between the proximal box and the access cavity could be possible. On the other hand in the traditional class II proximal-occlusal cavity group, the objective was to mimic the clinical scenario of endodontic treatment due to deep interproximal caries that have been reported frequently in the literature (20). Therefore, choosing between these designs by the clinicians depends mostly on the clinical situation of the teeth intended to be treated.

To avoid different outcomes caused by different operators’ skills, all specimen preparation procedures were conducted by the same operator. Maxillary first premolar teeth were used in this study because of the uniformity in size, form, and shape (21). The size of the teeth can be further controlled through restricted variance in crown dimensions of the teeth within 5% of the determined mean (22). The anatomical crown form of maxillary first premolars facilitates the cusp fractures under occlusal loads, as evidenced by the increased incidence of cusp fractures of upper premolars in the oral cavity (23). Moreover, cusp deflection is more common in maxillary premolars than in other posterior teeth, their anatomical shape, crown volume, and crown/root proportion may contribute to this complication (24).

The intercuspal measurements were performed by using a digital microscope (as a non-destructive method to take images for samples). This provides a detailed, easy, and reliable procedure facilitating storage and recall of the deflection data of the cusps. Also, this procedure assesses the liner deflection without any contact with the tooth, so it cannot interfere with the free movement of cusps compared to other techniques conventionally used to measure intercuspal distance such as the conventional caliper (25).

Different studies had been published in the literature regarding cusp deflection of teeth restored with bulk-fill composite resins (26-30); some of them have found a significantly less cusp deflection of bulk-fill composites compared to conventional resin-based composites (26-28), while others have found no superiority of bulk-fill composites over FiltekTM P60 conventional resin-based composite (29). On the other hand, a study concluded that bulk-fill composites resulted in a significantly higher cusp deflection compared to low shrinkage FiltekTM LS composite (30). In comparison of these studies with our study, the current study’s cusp deflection values were lower than those previously recorded values (26-30), likely attributed to the variations in experimental methodology. To maximize cuspal movement due to polymerization shrinkage of resin composite, large MOD restorations have been prepared in the previous literature (31), however, the focus of this study was to evaluate how various access cavity designs affect tooth stiffness so that more conservative cavities had to prepared with less dental structure removed as could as possible.

In this study, all teeth in the experimental groups showed inward cuspal movement after cavity preparation, this might be due to the preexisting residual stresses in the sound tooth. The cause for these stresses is not clear. However, they could be resulted from the extraction and water storage before using or are normal in teeth (32). In common with the present study findings, González *et al*. in 2006 found that higher cusp deflection was recorded 15 minutes after the restoration of the cavities in each group (9). This could be because of the resin-based restoration remaining free radicals and double bonds continued to react. Also, there was higher cusp deflection in teeth accessed by traditional class II cavity design compared to conservative class II and both class I cavity designs (thus the first part of the hypothesis has been rejected) as the cusp deflection was directly related to the volume of the tooth structural loss. The larger the loss of tooth structure, the lower the tooth’s resistance to deflection (33), and when restored, it will require more resin composite, resulting in higher contraction forces (34).

The results of this study showed that there was no significant difference between cusp deflection after cavity preparation from the cusp deflection after thermocycling of all restored teeth indicating that the cusps position had returned to approximately the original position before cavity preparation. Thermocycling in this study aimed to simulate the thermal alterations in the oral cavity to better simulate the clinical setting (35), and to determine whether there was a subsequent cuspal relaxation and if it would return to its original position. Cusp relaxation is represented as an outward movement of the cusp as a result of hygroscopic expansion and water sorption (36).

In contrast with our study, the majority of previously published studies of bulk-fill cusp deflection did not simulate thermocycling aging (26-30) but some of them performed water storage for 24, 48 hrs, and one week respectively, and noticed the occurrence of cusp relaxation (27,29).

Because of its ease of use and low cost, fracture strength was tested using static compressive stress in a universal testing machine (37). However, the loading to fracture used *in vitro* studies does not correctly reflect intraoral situations where failures are caused mostly by fatigue. Axial cyclical fatigue tests, on the other hand, may not represent the whole root strain patterns for the complex chewing process (1). The results of this study showed that the fracture strength of teeth in the conservative and traditional class I cavity groups was not significantly different, this could be attributed to the fact that the presence of marginal ridges in both cavities enhances the ability of the tooth to maintain its fracture strength. Also, there was no significant difference in fracture strength of both classes I and the conservative class II cavity group. This result could be explained by the fact that the dentin bridge in the latter group was maintained and thus connected the buccal and lingual walls of the cavity, which preserve tooth resistance to fracture as close as that in class I. The absence of significant difference between the two class II proximal-occlusal tested groups could be attributed to the loss of the marginal ridges as the loss of marginal ridge integrity was found to produce a 46 % drop in tooth strength (2). Thus, the second part of the hypothesis has been accepted.

The comparison of the resistance of the fracture of teeth accessed with the conservative or traditional access cavities by some studies has found no significant difference (1,15), which is consistence with the findings in the current study. On the other hand, other studies found that endodontic cavity size reduction with conservative access enhanced the fracture strength of teeth when compared to those accessed with the traditional one, permitting residual dentin preservation (4,14). These discrepancies could be due to the type and number of teeth included. In Plotino *et al*. in 2017, maxillary and mandibular premolars and molars were selected (14). Also in Krishan *et al*. 2014, maxillary incisors, mandibular premolars, and molars were selected (4). Other factors could have attributed to opposite results in this study compared to previous studies such as the application of restorations, the type of material used for restorative procedures, as well as methodological variances in the design of the fracture test (such as the direction of the applied load and size of tip used to induce fracture) (1,4,14,15).

 It is important to examine the fracture type in addition to fracture strength of endodontically-treated teeth as the non-restorable fracture in the dental structure necessitates tooth extraction (38). According to this study, intact teeth were associated with more restorable fractures than endodontically-treated groups, with less serious fracture patterns in teeth with conservative compared to traditional access cavity designs Table 2.

## Conclusions

Within the limitations of this study, it can be concluded that increasing tooth cavity preparation size including marginal ridge involvement maximizes the cusp deflection during restorative procedures. However, cusp deflection didn’t differ in class I conservative vs traditional cavities but it was higher in traditional class II vs conservative class II cavities. Also, conservative endodontic cavities may enhance the fracture resistance of endodontically-treated premolars but not to a statistically significant point in comparison to their traditional counterparts.
